# Causes of the Tendency to Use Common Traditional Therapies Among Older People in Miandoab, Iran: A Cross‐Sectional Study

**DOI:** 10.1002/hsr2.72647

**Published:** 2026-06-10

**Authors:** Abdolrasoul Safaeeian, Roghayyeh Armand, Hossein Matlabi

**Affiliations:** ^1^ Department of Epidemiology and Biostatistics Faculty of Health Sciences Tabriz University of Medical Sciences Tabriz East Azerbaijan province Iran; ^2^ Department of Health Education and Promotion Faculty of Health Sciences Tabriz University of Medical Sciences Tabriz East Azerbaijan province Iran; ^3^ Research Centre for Integrative Medicine in Aging, Aging Research Institute Tabriz University of Medical Sciences Tabriz East Azerbaijan province Iran; ^4^ Department of Geriatric Health Faculty of Health Sciences Tabriz University of Medical Sciences Tabriz East Azerbaijan province Iran

**Keywords:** herbal therapy, Iran, older people, tendency to use, traditional treatments

## Abstract

**Background and Aims:**

The increasing aging population in Iran, coupled with the prevalence of chronic diseases, has resulted in heightened attention to traditional medicine. This study aimed to examine the prevalence and factors influencing the use of traditional medicine among older adults in Miandoab, Iran.

**Methods:**

A cross‐sectional study was conducted with 450 participants, selected through a cluster sampling method. Data were collected using a structured questionnaire and analyzed with *t*‐tests, one‐way ANOVA, and Chi‐square tests.

**Results:**

A large majority (81.6%) of participants reported using traditional medicines. The most commonly used herbal remedies were mint (46.4%), cinnamon (42.5%), and ginger (41.8%). Furthermore, 39.8% and 47.1% of respondents indicated they would consult a bonesetter for a fracture or dislocation, respectively. Over one‐third of participants preferred traditional treatments as their initial option. The primary factor influencing this preference was word‐of‐mouth recommendations from previous generations (51.4%). Affordability and fewer perceived side effects compared to conventional medicine were the most frequently cited reasons for use. Women, individuals with lower educational attainment, and unemployed participants were more likely to initially select traditional treatments.

**Conclusion:**

The high reliance on traditional medicine, primarily due to cost and safety perceptions, highlights the need for healthcare policies that address affordability. Health insurance organizations should enhance financial support for vulnerable groups, including women, individuals with lower education and income, and those without supplementary insurance.

## 1 Introduction

The proportion of individuals aged 60 and over in Iran is projected to double from 10% in 2020 to over 20% by 2050, significantly increasing the burden of chronic age‐related conditions. National studies indicate that over 70% of older Iranians suffer from at least one chronic disease, such as hypertension or diabetes, which often requires long‐term management [1]. Although the prevalence and incidence of most chronic diseases are strongly age dependent, only 23% of the disability burden caused by chronic disease in countries with low and middle incomes occurs in people aged 60 years and older, compared with 36% for high‐income countries, where demographic ageing is much more advanced [2].

According to the Global Burden of Disease report, the five leading contributors to YLD (years of healthy life lost due to disability) among older people in low‐ and middle‐income countries are eye diseases, hearing loss, dementia, musculoskeletal diseases, and heart disease. Contributions of health problems to YLD are determined by their incidence, duration, and a disorder‐specific disability weight [3].

Older adults are the highest consumers of healthcare services [4, 5, 6]. Evidence shows that treatment costs for those over 65 are more than five times higher than for those under 65 [7]. These costs are both direct and indirect, with the latter including impacts on pension funds and the loss of experienced workers from the workforce [8, 9].

Since 2002, the World Health Organization has defined and recommended the integration of traditional and complementary medicine into national health systems, outlining a strategic plan for this purpose [10]. Older adults often turn to traditional, complementary, and alternative medicine due to dissatisfaction with conventional care, including poor patient‐provider relationships, limited consultation time, and the perception that natural products are safer and promote better health. Other reasons include the desire for more control over health decisions, relief from chronic symptoms, and the influence of peer pressure and advertising [11].

In recent decades, Iran has actively worked to revive and integrate its historically rich traditional medicine into the national healthcare system [12]. This coincides with a documented rise in interest in Traditional, Complementary, and Alternative Medicine (TCAM) among the Iranian public and medical professionals, with practices like herbal medicine, cupping, and acupuncture being particularly prevalent [13, 14]. This trend is especially noticeable among older adults, who often turn to TCAM to manage health issues that conventional medicine does not fully resolve. A common driver of this use is the perception that TCAM is both safer and more effective than pharmaceutical alternatives [15, 16]. However, this growing reliance, especially when TCAM is used concurrently with prescription drugs, presents significant clinical concerns. These include the risk of adverse interactions and questions about its adequacy for treating complex medical conditions.

Given the high utilization of traditional medicine among older adults, this study aimed to examine the prevalence and factors influencing the use of traditional medicine among older adults in Miandoab, Iran.

## 2 Materials and Methods

### 2.1 Study Design and Population

This descriptive‐analytical cross‐sectional study was conducted among older adults living in the rural areas of Miandoab city. The sample size was estimated to be 373 using Krejcie and Morgan [17] table and Cochran's formula [18]. To compensate for potential dropouts, 450 participants were recruited, including a 20% oversample. A two‐stage sampling method was employed: first, 45 health centers were randomly selected as clusters from a total of 108 in Miandoab; second, 10 older adults were selected from each center using convenience sampling. The inclusion of the entire population guarantees a sample size adequate for statistical relevance.

### 2.2 Data Collection

We used an Iranian valid questionnaire adapted from existing studies [14, 15]. The reliability was reported with a high Cronbach's alpha coefficient of 0.88. The questionnaire was structured into the following five sections:

Demographic and Socioeconomic Status: Eleven items.

Underlying Diseases: Twelve items.

Knowledge and Belief: Two items.

Usage and Information: One item each on usage frequency, purpose, information sources, and the primary reason for using traditional treatments.

Causes of Tendency: Seventeen items grouped into seven dimensions: knowledge (1 item), attitude/belief (2 items), advertising (3 items), accessibility (6 items), cost (2 items), effectiveness (4 items), and side effects (4 items). All items in this section used a 5‐point Likert scale (from 1 = “very low” to 5 = “very high”). An average score was computed for each multi‐item dimension.

### 2.3 Procedure

Following approval from the Research Council and Ethics Committee of Tabriz University of Medical Sciences (IR.TBZMED. REC.1399.281), official permits were acquired. Health workers at the participating centers were then informed of the study's aims. Participants were recruited with the cooperation of these centers, and written informed consent was obtained from all individuals. In the case of illiterate older adults, the process was administered verbally.

### 2.4 Data Analysis

Initially, descriptive statistics (frequencies, percentages, means, and standard deviations) were computed to characterize the sample demographics and the research variables. Subsequently, the relationships between these variables were investigated using inferential statistical tests, namely *t*‐tests, One‐way ANOVA, and Chi‐square. All statistical analyses were performed using SPSS version 22, with a significance level set at *p* < 0.05.

## 3 Results

Most participants were women, married, unemployed, and illiterate, with a monthly income of less than 40 dollars. The majority were covered by Iranian health insurance, but only 0.6% had supplemental insurance. Hypertension was the most common condition, followed by hyperlipidemia, diabetes, and cardiovascular diseases. About one‐third rated their health as very good or excellent, though most considered it average. Most participants reported taking medication, with a statistically significant higher percentage among women. Hypertension, hyperlipidemia, and diabetes were the most common reasons for medication.

Most older adults reported using herbal medicines, mint (46.4%), cinnamon (42.5%), and ginger (41.8%) being the most frequent. Referral to bonesetters for fracture and dislocation treatment was reported by 39.8% and 47.1% of participants, respectively, with women consulting bonesetters significantly more often than men. The most commonly used traditional treatments were cupping with paste, burning wild rue seeds (Espand), herbal therapy, prayer writing, Chilke (resetting the coccyx bone), and Qaeekh (blowing in the nose). The frequency of specific herbal remedies is summarized in Table 1.

**Table 1:** Frequency of specific herbal remedies used by older adults in Miandoab, Iran.

Medicinal herb	Frequency	Percentage (%)
Mint (Nana)	202	46.4
Cinnamon	185	42.5
Ginger	182	41.8
Oils and fats of animal origin	167	38.4
Turmeric	145	33.3
Licorice (Shirin Bayan)	129	29.7
Honey (Asal)	127	29.2
Grape Molasses (Doushab)	105	24.1
Willow Blossom Water (Araq‐e Bidmeshk)	96	22.1
Alfalfa Water (Araq‐e Yonje)	94	21.6
Borage (Gol‐e Gav Zaban)	78	17.9
Lemon Balm Water (Araq‐e Badranjboya)	77	17.7
Mountain Savory (Marze Koohi)	58	13.3
Marshmallow Root (Gol‐e Khatmi)	54	12.4
Black Seed (Siah Dana)	53	12.2
Yarrow (Boomadaran)	53	12.2
Thyme (Avishan)	51	11.7
Fennel (Raziyane)	43	9.9
Wild Ice Plant (Khak Shir)	40	9.2
Four Seeds (Chahar Takhme) ‐ Seed Mix	35	8.0
Quince Seed (Takhme Beh)	34	7.8
Mallow (Panirak)	32	7.4
Grape Molasses (Doushab)	105	24.1
Alhagi (Khar‐e Shotor)	29	6.7
Fenugreek (Shanbalile)	27	6.2
Purslane (Kharfe)	25	5.7
Chamomile (Baboone)	22	5.1
Hibiscus Tea (Chay‐e Torsh)	21	4.8
Frankincense (Kondor)	21	4.8
Ziziphora (Kakuti)	20	4.6
Ghaz Ayaghi	17	3.9
Cumin (Zire)	16	3.7
Wild Rose (Gol‐e Nastaran)	16	3.7
Amaranth (Taj Khoros)	11	2.5
Vine Twig Smoke (Dood‐e Shakhe Tak)	10	2.3
Mountain Watermelon (Hendavane Koohi)	9	2.1
Mountain Tea (Chay‐e Koohi)	8	1.8
Jujube (Annab)	7	1.6
Barley Oil (Roghan‐e Jo)	6	1.4
Mumia (Mumiya)	6	1.4
Black Oil/Kerosene (Naft‐e Siah)	5	1.1
Unidentified	5	1.1

The initial use of traditional treatments upon sickness was more common among women, those with lower education, and unemployed individuals. Knowledge/belief, advertising, and effectiveness factors also contrasted significantly based on income, lower scores among participants earning above $200 per month (Table 2).

**Table 2:** Socio‐demographic status and preference of traditional or modern medicine treatments in response to disease symptoms.

Variables	Items	Traditional medicine	Modern medicine	*χ* ^2^	*p* value
Gender	Male	27.5	72.5	6.91	0.009
	Female	39.9	60.1		
Levels in education	Illiterate	39.4	60.6	12.54	0.01
	Elementary	26.7	73.3		
	Secondary	40.0	60.0		
	High School	7.1	92.9		
	Higher education	11.1	88.9		
Employment	Employed	26.9	73.1	5.02	0.02
	Unemployed	38.4	61.6		
Income (per month)	Less than $100	37.7	62.3	2.8	0.4
	$100‐$200	36.0	64.0		
	$200‐$300	28.6	71.4		
	More than $300	25.9	74.1		
Basic insurance	Covered	34.9	65.1	0.28	0.59
	Not covered	41.2	58.8		
Supplementary insurance	Covered	26.9	73.1	0.82	0.36
	Not covered	35.7	64.3		

Only 12.5% of participants reported high or very high frequency of traditional treatment use, 12.4% reported high familiarity, and 13.6% reported high belief. The average scores for use, familiarity, and belief were generally below average. Women had a significantly higher average score for using traditional treatments than men.

The most effective sources of information encouraging traditional treatment use were word‐of‐mouth from previous generations, consultation with family and friends, and customs/traditions. No significant gender differences were observed in these sources.

Men used traditional treatments more for maintaining health and disease prevention, while women used them more for relieving pain and symptoms (Table 3).

**Table 3:** Viewpoints of older people toward advantages of traditional treatments.

Aims	Frequency	Percentage
Be healthy and health promotion	103	25.3
Treatment of diseases	255	62.7
Relief of pain and symptoms	49	12

A significant difference was found between men and women regarding reasons for using traditional treatments. For women, the primary reasons were lower cost, positive advertisements, and a positive attitude; for men, the main reasons were fewer side effects and lower cost (Table 4).

**Table 4:** Gender and frequency of the most important reasons for using traditional treatments.

Reasons for using traditional treatments	Frequency	Percentage	Male	Female	*X* ^2^	*p*‐value
Attitude and belief	44	10.2	9.0	10.9	13.84	0.0.3
Advertisement	71	16.5	11.4	19.6		
Accessibility	44	10.2	13.9	7.9		
Cost	99	23.0	22.3	23.4		
Effectiveness	11	2.6	3.6	1.9		
Less side effects	90	20.9	25.9	17.7		

Analysis based on demographic variables showed that all factors influencing the tendency to use traditional treatments, except ease of access, were statistically different based on supplemental insurance status. Average scores for all factors were higher in participants without supplemental insurance. Knowledge/belief and advertisement factors showed a significant relationship with marital status, with married participants scoring higher than singles (Table 5).

**Table 5:** Mean and standard deviation of factors affecting the inclination to use traditional treatments based on supplementary insurance and marital status.

Factors	Marital status	Mean ± standard deviation	Z	P value	Supplementary insurance	Mean ± standard deviation	Z	*p* value
Attitude and belief	Single	38.76 ± 18.00	−2.45	0.01	Covered	29.80 ± 21.23	−2.95	0.003
	Married	43.75 ± 23.39			Not covered	43.18 ± 21.97		
Advertisement	Single	37.07 ± 22.81	−3.28	0.001	Covered	32.21 ± 19.09	−2.21	0.02
	Married	43.78 ± 22.01			Not covered	42.58 ± 22.48		
Accessibility	Single	24.22 ± 2.42	−0.55	0.58	Covered	24.67 ± 1.63	0.81	0.41
	Married	24.34 ± 2.34			Not covered	24.28 ± 2.4		
Cost	Single	35.14 ± 23.2	−1.31	0.18	Covered	28.00 ± 19.18	−2.11	0.03
	Married	37.98 ± 22.7			Not covered	37.77 ± 22.95		
Effectiveness	Single	32.63 ± 19.16	−1.6	0.1	Covered	23.00 ± 16.52	−3.04	0.002
	Married	36.49 ± 22.04			Not covered	36.27 ± 21.41		
Less side effects	Single	34.97 ± 22.37	−1.36	0.17	Covered	25.32 ± 19.07	−2.69	0.007
	Married	38.72 ± 24.31			Not covered	38.50 ± 23.9		

Participants with lower education levels had significantly higher average scores for knowledge/belief, advertising, and effectiveness factors compared to those with higher education, particularly at the high school diploma level and above. All factors except ease of access showed significant differences based on living conditions; those living alone had lower average scores across all factors (Table 6).

**Table 6:** Mean and standard deviation of factors affecting the inclination to use traditional treatments based on educational levels and living arrangements.

Factors	Education level	Mean ± standard deviation	F	*p* value	Living arrangements	Mean ± standard deviation	F	*p* value
Attitude and belief	Illiterate	43.25 ± 20.61	8.87	0.01	Alone	28.57 ± 15.19	42.79	< 0.0001
	Reading and Writing	43.4 ± 23.5			Spouse	33.21 ± 18.36		
	High school and more	26.08 ± 28.68			Spouse and children	50.15 ± 20.01		
Advertisement	Illiterate	41.45 ± 21.55	11.43	0.003	Alone	28.27 ± 15.63	18.53	< 0.0001
	Reading and Writing	45.64 ± 24.37			Spouse	37.32 ± 21.92		
	High school and more	30.97 ± 20.25			Spouse and children	47.00 ± 22.17		
Accessibility	Illiterate	24.31 ± 2.39	0.58	0.74	Alone	24.2 ± 2.47	0.28	0.75
	Reading and Writing	24.24 ± 2.4			Spouse	24.42 ± 2.11		
	High school and more	24.16 ± 3.73			Spouse and children	24.25 ± 2.49		
Cost	Illiterate	39.99 ± 22.65	2.02	0.36	Alone	28.57 ± 16.39	6.95	0.001
	Reading and Writing	38.87 ± 23.28			Spouse	34.17 ± 21.8		
	High school and more	32.06 ± 23.48			Spouse and children	40.49 ± 23.83		
Effectiveness	Illiterate	35.99 ± 21.17	7.17	0.02	Alone	27.00 ± 14.22	8.64	< 0.0001
	Reading and Writing	36.25 ± 21.51			Spouse	31.99 ± 22.6		
	High school and more	25.00 ± 21.45			Spouse and children	39.05 ± 20.91		
Less side effects	Illiterate	34.48 ± 23.81	3.14	0.2	Alone	30.00 ± 17.06	5.18	0.006
	Reading and Writing	37.07 ± 23.88			Spouse	34.78 ± 24.16		
	High school and more	30.43 ± 23.51			Spouse and children	40.7 ± 24.16		

## 4 Discussion

This study found that cupping with paste, burning wild rue, herbal therapy, prayer writing, Chilke, and Qaeekh were the most commonly used traditional treatments. This highlights the need to evaluate the efficacy of these practices and publicize findings, especially for ineffective treatments. Similar studies reported high use of herbal medicines and spiritual therapies [19, 20].

Mint, cinnamon, and ginger were the most frequently used herbs, reflecting the long‐standing cultural practice of using medicinal plants in Iran [13]. A significant proportion of participants would consult bonesetters for fractures (39.8%) and dislocations (47.1%), with women doing so more frequently than men.

In rural Miandoab, traditional medicine is a significant, culturally ingrained practice, particularly among socioeconomically vulnerable groups. A significant proportion, exceeding 36%, of older adults, especially women, the less educated, and the unemployed, prefer it as a first resort. This suggests that higher socioeconomic status may correlate with greater use of modern healthcare. The primary motivations are cost‐effectiveness and the perception of fewer side effects compared to conventional medicine. The high cost of modern medical care, despite insurance, remains a major driver [12, 15].

In an Ethiopian study titled “use of complementary and alternative medicine among older adults suffering from chronic diseases,” the results indicated that dissatisfaction with conventional treatment (40.8%) and belief in the effectiveness of complementary and alternative therapies (30.8%) were the most common reasons for using complementary and alternative medicine. Moreover, 74% of the older people reported using complementary and alternative medicine, and the use of herbal medicines (50.4%) and spiritual healing (40.8%) were the most common methods. The highest frequency of herbal medicine use was related to mint, cinnamon, and ginger, respectively. Rural location, higher educational status, higher average monthly income, and the presence of co‐morbidities were positively associated with the use of complementary and alternative medicine [19, 21].

The use of traditional treatments is deeply rooted in cultural transmission, with family and word‐of‐mouth being the primary sources of information. While only 3.5% of participants were regular users, most had tried them at least once in the past year, indicating a widespread, general belief in their value. The main reason for use was symptomatic relief. A gender difference reported as women focused more on relieving pain, while men were more inclined towards health maintenance [13].

In a Korean study titled “complementary and alternative medicine use behaviors and affecting factors amongst older people” in 2013, the results showed that 70.4% of the older adults had used various types of complementary and alternative medicine, including nutritional methods, pharmacological and biological treatments, more than once in the past year. Interestingly, 48.8%–60.7% of the participants used complementary therapies without consulting their physicians or pharmacists. Also, having chronic diseases increases the likelihood of using complementary and alternative medicine [10, 11].

A further study titled “assessment of knowledge, attitude and practice of older adults toward complementary and alternative medicine referring to health insurance outpatient clinics in Ismailia province, Egypt” in 2018, revealed that about 84% of the participants had sufficient knowledge and a positive attitude towards complementary and alternative medicine. Moreover, their source of knowledge was mainly the media (56%), followed by their family and friends. Finally, 76% believed that complementary and alternative medicine was safe and 58.7% believed that it was effective [7].

Socioeconomic factors are strongly linked to usage patterns. Individuals with lower education and income, as well as those without supplemental insurance, demonstrated a stronger belief in traditional treatments and were more influenced by advertisements [22, 23]. Larger household size was also a correlating factor, likely due to lower per‐capita income and education [24]. Interestingly, the perceived ease of access to traditional treatments was uniformly high across all demographic groups and was not a differentiating factor. The highest‐scoring influence was deeply‐held knowledge and belief, confirming that the tendency is fundamentally cultural rather than merely a matter of convenience [24, 25].

## 5 Conclusions

To conclude, the widespread use of traditional treatments among the elderly in rural Miandoab is driven by cultural transmission, cost‐effectiveness, and a perception of greater safety compared to conventional medicine. These practices are most prevalent among vulnerable demographics, including women and individuals with lower socioeconomic status.

This trend presents significant public health challenges. Key concerns include the potential for adverse drug interactions and the risk that dependence on traditional medicine could result in the underutilization of essential conventional healthcare services.

Based on the findings, the following recommendations are proposed:

1. It is essential to develop and distribute clear, accessible education about common traditional treatments through health centers. This initiative should specifically consider local literacy rates and prioritize reaching women.

2. Healthcare providers should routinely inquire about the use of traditional treatments to identify and manage potential drug‐herb interactions when prescribing conventional medicines.

3. Policymakers should review the affordability of conventional medical treatments to ensure that high costs do not force individuals to opt for traditional alternatives.

4. Health insurance organizations should enhance financial support and coverage for vulnerable groups, including women, individuals with low income and education, and those without supplementary insurance, to improve their access to conventional medical care.

## Limitations

The reliance on convenience sampling methods introduces a substantial risk of selection bias, compromising the study's external validity. Consequently, the generalizability of the results is limited to the particular subgroup from which the sample was selected, rather than the population at large.

## Author Contributions

**Abdolrasoul Safaeeian:** software, methodology, validation, formal analysis. **Roghayyeh Armand:** writing – original draft, resources. **Hossein Matlabi:** conceptualization, investigation, methodology, supervision, project administration, writing – review and editing, writing – original draft.

## Funding

The authors received no specific funding for this work.

## Disclosure

The lead author Hossein Matlabi affirms that this manuscript is an honest, accurate, and transparent account of the study being reported; that no important aspects of the study have been omitted; and that any discrepancies from the study as planned (and, if relevant, registered) have been explained. All authors have read and approved the final version of the manuscript. Hossein Matlabi had full access to all of the data in this study and takes complete responsibility for the integrity of the data and the accuracy of the data analysis.

## Ethics Statement

The study was approved by the Ethics and Research Committee of Tabriz University of Medical Sciences (IR.TBZMED.REC.1399.281). Informed consent was obtained from all individual participants.

## Conflicts of Interest

The authors declare no conflicts of interest.

## Data Availability

The data that support the findings of this study are available from the corresponding author upon reasonable request.
